# Intra-specific Niche Partitioning in Antarctic Fur Seals, *Arctocephalus gazella*

**DOI:** 10.1038/s41598-020-59992-3

**Published:** 2020-02-24

**Authors:** Kayleigh A. Jones, Norman Ratcliffe, Stephen C. Votier, Jason Newton, Jaume Forcada, John Dickens, Gabriele Stowasser, Iain J. Staniland

**Affiliations:** 1 British Antarctic Survey, Cambridge, United Kingdom; 2 University of Exeter, Exeter, England; 3 Scottish Universities Environmental Research Centre, East Kilbride, Scotland

**Keywords:** Biological techniques, Ecology, Zoology

## Abstract

Competition for resources within a population can lead to niche partitioning between sexes, throughout ontogeny and among individuals, allowing con-specifics to co-exist. We aimed to quantify such partitioning in Antarctic fur seals, *Arctocephalus gazella*, breeding at South Georgia, which hosts ~95% of the world’s population. Whiskers were collected from 20 adult males and 20 adult females and stable isotope ratios were quantified every 5 mm along the length of each whisker. Nitrogen isotope ratios (δ^15^N) were used as proxies for trophic position and carbon isotope ratios (δ^13^C) indicated foraging habitat. Sexual segregation was evident: δ^13^C values were significantly lower in males than females, indicating males spent more time foraging south of the Polar Front in maritime Antarctica. In males δ^13^C values declined with age, suggesting males spent more time foraging south throughout ontogeny. In females δ^13^C values revealed two main foraging strategies: 70% of females spent most time foraging south of the Polar Front and had similar δ^15^N values to males, while 30% of females spent most time foraging north of the Polar Front and had significantly higher δ^15^N values. This niche partitioning may relax competition and ultimately elevate population carrying capacity with implications for ecology, evolution and conservation.

## Introduction

Competition for resources within a natural population can lead to diversification in resource use, ultimately allowing con-specifics to co-exist^1^. The ecological niche is positioned within an *n*-dimensional hypervolume^2^, generally composed of spatial, temporal and trophic axes^3^. Overlap in ecological niches causes competition for resources, which could lead to competitive exclusion^4,5^ and consequent niche shifts, whereby the position of a niche alters along the spatial, temporal, and/or trophic axis^6,7^. This niche partitioning commonly arises between sexes, but can also occur throughout ontogeny (hereby over an organism’s lifespan) and among individuals within a species^8^. The consequent reduction in intra-specific competition may lead to a greater carrying capacity for the population as a whole^9–11^. Understanding the causes and consequences of intra-specific niche partitioning is therefore a major goal of research into the ecology, evolution and conservation of species^12,13^.

Niche partitioning between sexes has been explained by several inter-connected hypotheses: (1) social roles: sexes segregating because they prefer to associate with the same class to benefit from social learning^14,15^; (2) activity budgets: sexes segregating to synchronise activities (e.g. sex-specific behaviours) to enable spatial coherence of the social group^16,17^; (3) life history strategies: including constraint of parental care; and (4) sexual size dimorphism (common in species with polgynous mating systems^18^). The sexual size dimorphism hypothesis has received considerable attention as body size is a key trait influencing fitness^19^. Indeed, males with larger body sizes could compete for mates more successfully^20^. Smaller animals can subsist on sparser resources than larger animals^21,22^, but may require higher quality food because of their higher mass-specific metabolic rates^23^. For example, adult female African elephants, *Loxodonta africana*, and their offspring feed in areas with greater plant diversity than larger adult males, which are less selective^24^. Size dimorphism also affects susceptibility to predation^25,26^ and physiological constraints (such as temperature and aerobic dive limits in diving predators^27^).

The above hypotheses relate to animals differently throughout ontogeny. As animals grow their life history priorities change, from maximising growth and survival as juveniles^28^ to reproduction as adults^29,30^. Ontogenetic niche shifts may occur as animals grow, become sexually mature and gain more experience with age. They could differ between the sexes, as a result of different life history constraints that affect growth patterns and resulting sexual-size dimorphism. Ontogenetic niche shifts may be particularly pronounced in the larger sex, as larger animals experience a greater diversity of body sizes (and therefore energetic requirements) throughout development^3^. For example, Northern death adders, *Acanthopis praelongus*, predate on frogs and lizards as juveniles, and frogs and mammals as adults, but adult females (the larger sex) consume a greater proportion of mammals than adult males^31^.

Niche differentiation can also occur among individuals, when individuals occupy only a subset of the population’s niche (individual specialisation)^32^. The optimum strategy for an individual depends on its particular priorities and restraints^33^. Individuals may rank resources differently according to their energy gain per unit time^32^ because of their size, age and experience, which affect diet preference, search efficiency and prey handling ability^34,35^. Different foraging strategies may therefore develop within the same sex. For example, female New Zealand sea lions, *Phocarctos hookeri*, have three distinct foraging strategies – a mechanism which could reduce intra-specific competition^36^.

Intra-specific niche partitioning may influence population carrying capacity. Theory on habitat selection predicts that as population density and competition increases, animals should distribute themselves relative to habitat profitability^37^. Selection favours behavioural and morphological traits that reduce aggressive encounters and competition for resources^38,39^. Individuals may specialise on particular resources^40^ and the population as a whole may exploit a wider range of resources^37^. For example, a population of feral horses, *Equus ferus caballus*, use a greater diversity of resources as population density increases^37^. It is therefore possible that generalist populations are composed of both generalist and specialist individuals^40^. These mechanisms reduce competition, which could increase individual reproductive success and consequently elevate population carrying capacity.

**Figure 1:**
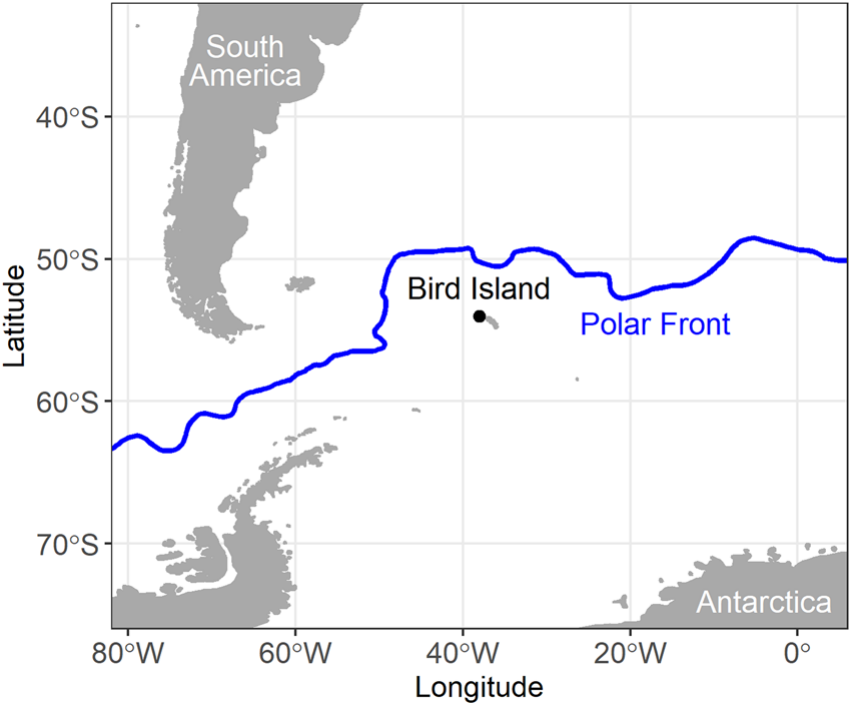
Map showing location of Bird Island, South Georgia, in relation to South America, Antarctica and the Polar Front. Map was created using R software (v3.6.1; https://www.R-project.org/).

Antarctic fur seals, *Arctocephalus gazella*, are an ideal species to study intra-specific niche partitioning because of their large population size, breeding constraints and pronounced sexual size dimorphism (related to a highly polgygynous mating system). Intra-specific competition may be intense as populations have recovered from near extinction and are now in their millions with ~95% breeding at South Georgia, situated in the southern Atlantic Ocean^41^ (Fig. 1). The sexes have different breeding constraints, as females arrive at breeding beaches in late November/early December and are spatially restricted for four months while alternating foraging at sea with suckling their pups^27^. Males come ashore from October (peaking in numbers in December), to establish and defend harems (territorial males may fast at this time)^41^. After mating, males have no spatial or temporal constraints and observations suggest they migrate to higher latitudes in January^27,41–43^. Short-term tracking of individual females has shown that they migrate widely in winter, moving north towards Patagonia, south towards the Antarctic pack-ice and within waters around South Georgia^44–46^. However, it is unknown if these movement patterns are consistent across years and/or individuals.

The sexes have different growth trajectories that may facilitate ontogenetic niche shifts: females reach 90% of their maximum body length and become sexually mature by age four, while males grow to age seven (weighing up to four times more than females) and may not establish territories until age seven or eight^41,47^. Niche partitioning between sexes and throughout ontogeny has been determined in Antarctic fur seals breeding at Kerguelen^48^. However, Antarctic fur seals breeding at South Georgia may show different patterns in niche partitioning as a result of different environments, diets (i.e. Antarctic krill, *Euphausia superba*, predominates the diet at South Georgia^41^, while myctophids dominate at Kerguelen^49^), and the higher competitive pressure associated with a higher population density.

Stable isotope analysis can provide quantitative insights into intra-specific niche partitioning^48,50^ as stable isotope values are represented in delta-space as the ‘isotopic niche’^51^. Stable isotope values of a consumer’s tissues in part reflect diet, plus an added trophic discrimination factor (TDF) signifying the offset in stable isotope values between the tissue and the consumer’s food^52^. This offset occurs as a result of physical and biological processes involved in assimilating resources^53^. Nitrogen isotope ratios (^15^N/^14^N expressed as δ^15^N) are used as a proxy for trophic position, as the ratio increases stepwise with trophic level^54,55^. In marine systems carbon isotope ratios (^13^C/^12^C expressed as δ^13^C) indicate the geographic source of prey as they vary with offshore versus inshore regions, pelagic versus benthic regions, and notably latitude^56,57^. The δ^13^C values in particulate organic carbon in the oceans generally decline from the tropics to the poles^58,59^ and can be distinct between water masses separated by frontal zones: a pattern reflected by δ^13^C values of marine predators in sub-Antarctic regions^60–64^.

To investigate the existence and development of intra-specific niche partitioning and its role in reducing competition we analysed δ^15^N and δ^13^C values along the length of adult Antarctic fur seal whiskers. These are ideal tissues to study ontogeny and individual specialisation as they are metabolically inert once formed, grow continuously, and are retained for years so can reflect the animal’s foraging over long time periods. We hypothesise that for Antarctic fur seals within the world’s largest breeding colony: (1) Males will have lower δ^13^C values along their whiskers as they spend more time in maritime Antarctica than females; (2) Males will show greater ontogenetic changes in their isotopic niche as they exhibit greater growth than females and do not breed until they are older; (3) Females will show greater variation in δ^15^N values and δ^13^C values, reflecting a wider range of post-breeding migration strategies; (4) Consistent annual patterns in isotopic values will show that these migration strategies are consistent between years.

## Results

### Seal age

Average age of adult males, obtained from external growth ridges in canines, was 8.94 ± 0.89 years for all 34 males and 8.70 ± 0.73 for 20 randomly selected males. These age determinations were in close agreement between any two readers, with 92.1% of all readings showing a 0 or ±1 year difference. Age determinations were fairly consistent among all three readers, as the Index of Average Percentage Error (IAPE) was relatively low at 4.3%. Average minimum age of adult females at capture was 7.45 ± 2.17 years according to whisker growth rates.

### Whisker growth rates

**Figure 2:**
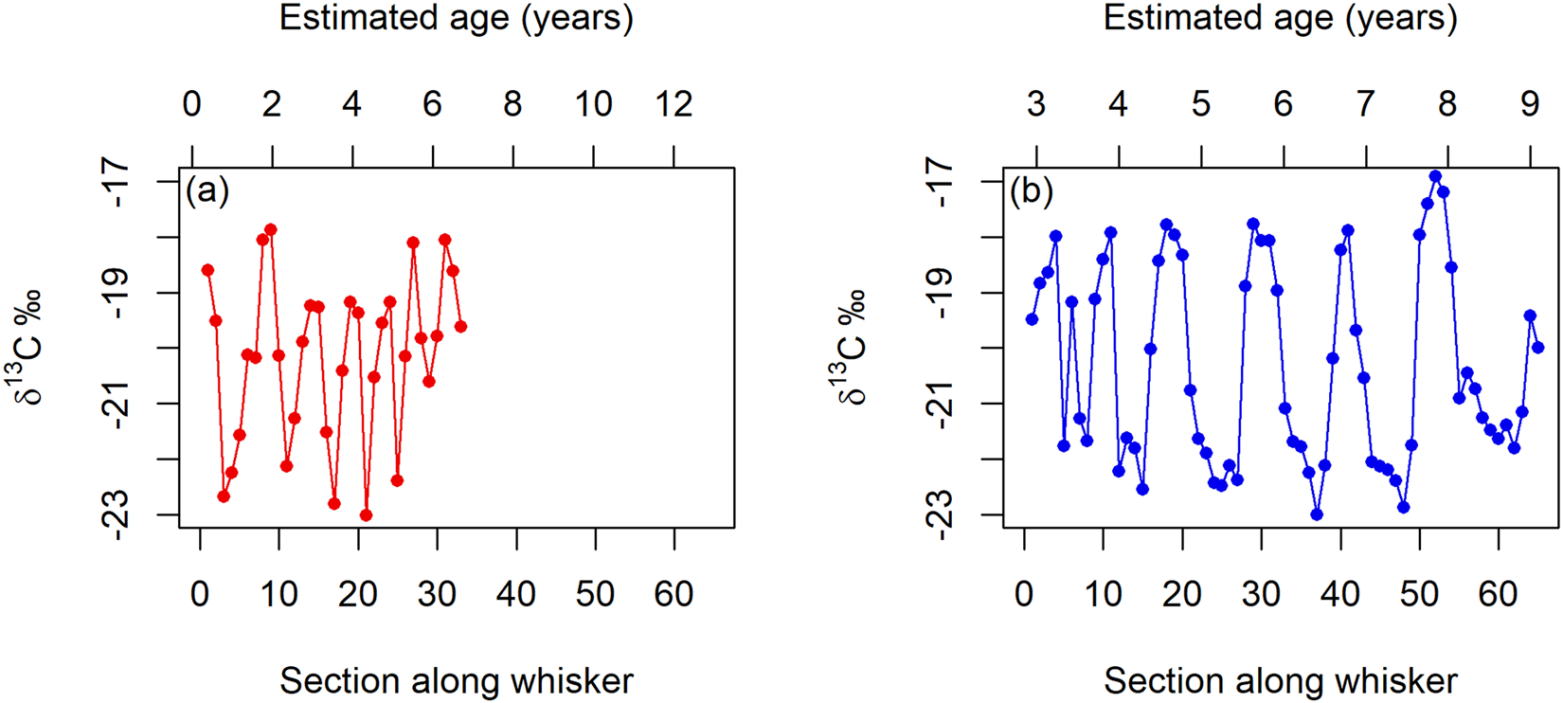
Oscillations in δ^13^C values along the length of (**a**) a female Antarctic fur seal whisker (ID = w8859) and (**b**) a male Antarctic fur seal whisker (ID = w8821) from the distal to the facial end. Points are δ^13^C values of samples taken every 5 mm along the length of each whisker and lines join these points. Figure was created using R software (v3.6.1; https://www.R-project.org/).

Whisker length significantly differed between males (mean = 25.75 cm, SD = 6.95) and females (mean = 16.29 cm, SD = 4.53, excluding the whisker root ~0.5 cm in length) (Mann-Whitney U test, U = 42, p < 0.001). Male whiskers also grew significantly faster (0.096 ± 0.026 mm/day) than female whiskers (0.063 ± 0.013 mm/day), assuming oscillations corresponded to annual migrations (Welch’s t-test, t = 5.29, p < 0.001) (e.g. Fig. 2; Supplementary Fig. S1). The calculated whisker growth rates suggest that male whiskers were grown over an average of 6.93 ± 2.03 years and females an average of 7.18 ± 1.20 years.

### δ^13^C value of polar front

**Figure 3:**
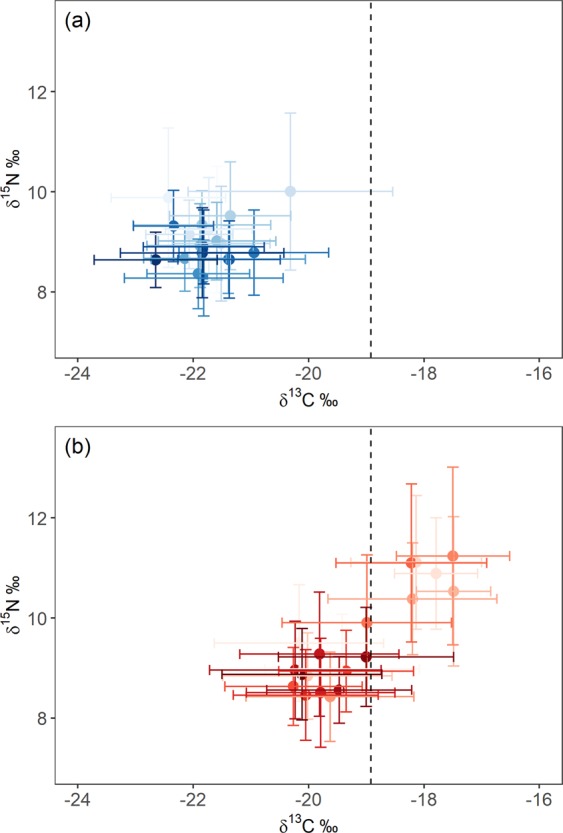
Bi-plots showing the means (points) and standard deviations (lines) of δ^13^C and δ^15^N values in whiskers of (**a**) 20 male and (**b**) 20 female Antarctic fur seals breeding at South Georgia. Dashed line indicates estimated δ^13^C value of whiskers when seals foraged at the Polar Front. Figure was created using R software (v3.6.1; https://www.R-project.org/).

When seals foraged at the Polar Front we estimated that δ^13^C values in their whiskers were about −18.92‰. This value was calculated from the average δ^13^C value of prey species (myctophids and krill) collected at the Polar Front in 2009 (−20.98‰) (Supplementary Fig. S2), added to the estimated TDF for Antarctic fur seal whiskers (2.06‰ ± 1.79 for δ^13^C). The variation in δ^13^C values along each whisker suggested all 20 females and only six males foraged north of the Polar Front at any point during their lives (as their maximum δ^13^C values exceeded −18.92‰) (Supplementary Tables S3 and S4). Stable isotope bi-plots (Fig. 3) revealed two isotopically distinct groups of females, separated by the estimated δ^13^C value of whiskers at the Polar Front: 14 individuals (female Group 1) had lower mean δ^13^C values (using all δ^13^C values along the whisker) than −18.92‰ and 6 individuals (female Group 2) had higher mean δ^13^C values than −18.92‰.

### Sex-specific niche partitioning

**Figure 4:**
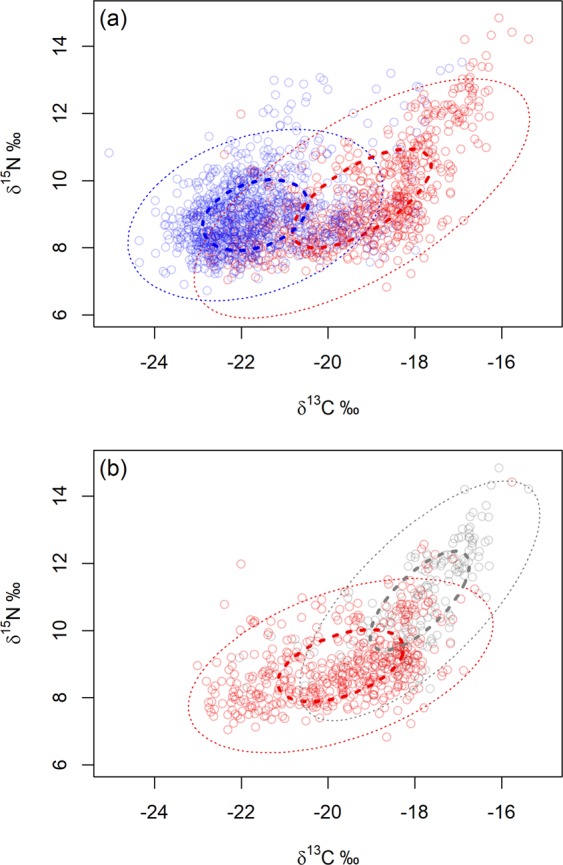
Standard Ellipse Areas (SEAs) representing the isotopic niches of (**a**) 20 male (blue) and 20 female (red) Antarctic fur seals by δ^13^C and δ^15^N values in their whiskers and (**b**) isotopic niches of females according to the estimated δ^13^C value of whiskers at the Polar Front (−18.92‰): female Group 1 (red) consists of 14 individuals with mean δ^13^C values below −18.92‰; female Group 2 (grey) consists of 6 individuals with mean δ^13^C values above −18.92‰. Points are isotopic values of each whisker sample, bold dashed ellipses use 40% of data points and dotted ellipses use 95% of data points. Figure was created using R software (v3.6.1; https://www.R-project.org/).

Sexual segregation occurred primarily in foraging distribution (along the carbon axis), but not in trophic position (along the nitrogen axis). Mean δ^13^C values were substantially lower in males than females (−21.68‰ ± 1.20 and −19.22‰ ± 1.58 respectively), while mean δ^15^N values were similar in males and females (8.98‰ ± 1.04 and 9.47‰ ± 1.45 respectively). Females occupied a larger isotopic niche than males as Standard Ellipse Areas (SEAs) were 5.39 for females (Bayesian Standard Ellipse Area (SEA_*B*_) mode: 5.49 with 95% credibility interval 5.00–5.82), and 3.72 for males (SEA_*B*_ mode: 3.83 with 95% credibility interval 3.50–3.96) (Fig. 4a). Isotopic niches were distinct between the sexes, as male and female SEAs only overlapped by 1.2% (1.1% using Bayesian inference with 95% credibility interval 0.089–2.37).

### Isotopic differences within females

Female Group 1 mainly foraged at higher latitudes on potentially lower trophic level prey than female Group 2, as both mean δ^15^N and δ^13^C values were lower in female Group 1 than female Group 2 (means of each group: 8.96‰ ± 1.06 and 10.89‰ ± 1.46 respectively for δ^15^N; −19.71‰ ± 1.44 and −17.89‰ ± 1.13 respectively for δ^13^C). Female Group 1 occupied a slightly larger isotopic niche than female Group 2 as SEAs were 4.21 for female Group 1 (SEA_*B*_ mode: 4.20 with 95% credibility interval 3.85–4.58) and 3.61 for female Group 2 (SEA_*B*_ mode: 3.62 with 95% credibility interval 3.11–4.21). These female groups were largely distinct as SEAs overlapped by only 4.5% (3.1% using Bayesian inference with 95% credibility interval 1.01–9.17) (Fig. 4b). Males likely competed more with female Group 1 (SEAs overlapped by 5.1%; 4.8% using Bayesian inference with 95% credibility interval 3.26–6.77) than female Group 2 (SEAs did not overlap).

### Body size differences within females

Females in Group 1 were significantly smaller than females in Group 2, as indicated by principal components analysis (PCA). Specifically, loadings for principal component 1(PC1) were highest for mass (−0.57), span (−0.52), length (−0.50) then girth (−0.40), while loadings for principal component 2 (PC2) were highest for girth (0.84), length (−0.47), span (−0.25) then mass (0.04). PC1 and PC2 explained 18.2% and 73.9% of variability in morphology data respectively. The mean scores between the two female groups differed by 1.97 for PC1 (Welch’s t-test: t = −2.70, p = 0.02) and 0.98 for PC2 (Welch’s t-test: t = 3.11, p = 0.01) (Supplementary Fig. S5).

### Ontogeny of sexual segregation in isotopic niche

**Figure 5:**
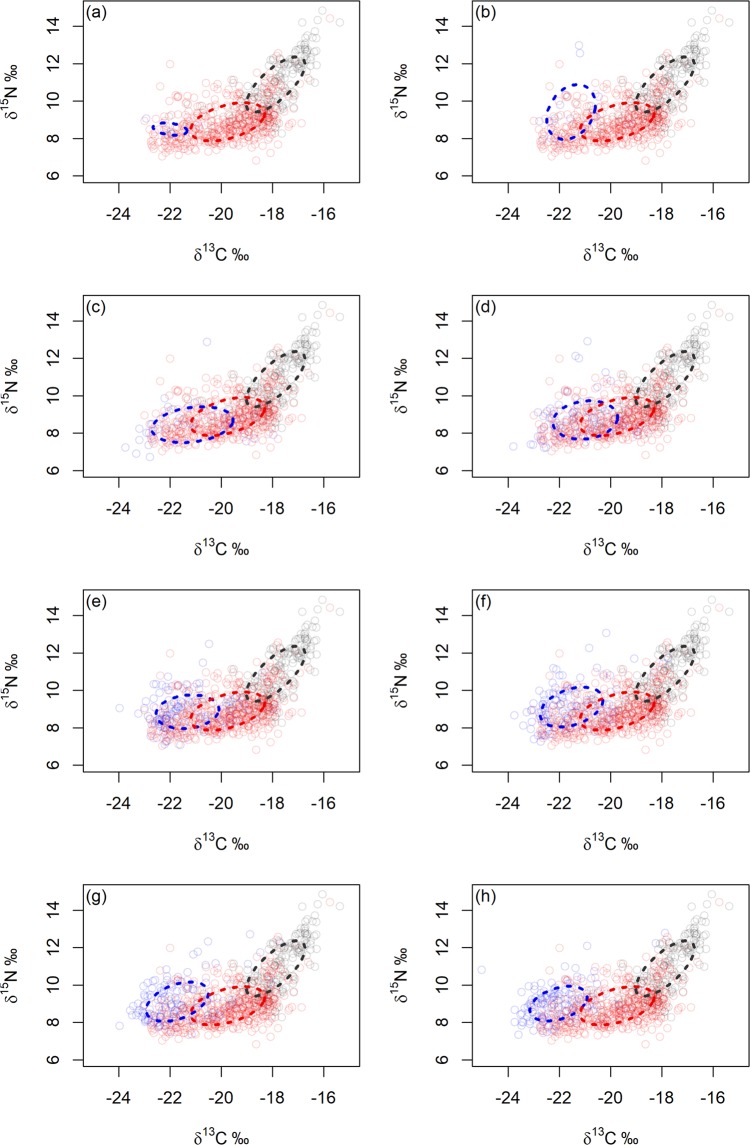
The ontogeny in isotopic niche of male Antarctic fur seals (blue) as they age compared to Standard Ellipse Areas (SEAs) of female Group 1 (red) and female Group 2 (grey). Males are aged (**a**) 0.5–1 year; (**b**) 1–2 years; (**c**) 2–3 years; (**d**) 3–4 years; (**e**) 4–5 years; (**f**) 5–6years; (**g**) 6–7 years; (**h**) 7–8 years. Points are isotopic values of each whisker sample and bold dashed ellipses represent SEAs using 40% of data points for each group: blue represents the isotopic niche of males; red SEA represents the overall isotopic niche of female Group 1 (females with lower mean δ^13^C values than estimated δ^13^C value of whiskers at the Polar Front) and grey SEA represents the overall isotopic niche of female Group 2 (females with lower mean δ^13^C values than estimated δ^13^C value of whiskers at the Polar Front). Figure was created using R software (v3.6.1; https://www.R-project.org/).

**Figure 6:**
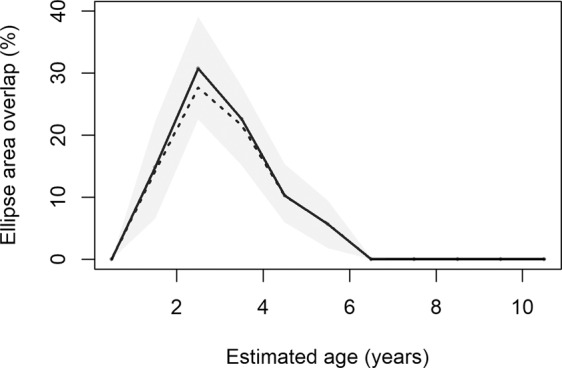
Percentage overlap in Standard Ellipse Area (SEA) of male Antarctic fur seals as they age with female Group 1 (females with lower mean δ^13^C values than estimated δ^13^C value of whiskers at the Polar Front). Bold line shows overlap in SEA using maximum likelihood, dashed line shows mode overlap using Bayesian inference, and grey shaded region shows 95% credibility interval around this mode. Figure was created using R software (v3.6.1; https://www.R-project.org/).

Ontogenetic niche shifts were present in males on an annual scale. SEA increased between ages 0.5–2 years (although there were only 6 samples for males aged 0.5–1), then generally declined with age thereafter (Fig. 5; Supplementary Table S6). Overlap in isotopic niche between males of different age classes and SEA of female Group 1 increased from males aged 0.5–3 years, then declined with increasing male age until only 0.0015% of overlap occurred when males aged 6–7 years and no overlap in SEA occurred thereafter (Fig. 6; Supplementary Table S7). No overlap in SEA occurred between any male age group and female Group 2.

### Contributions of sex, age and individual to isotopic niche differentiation

**Figure 7:**
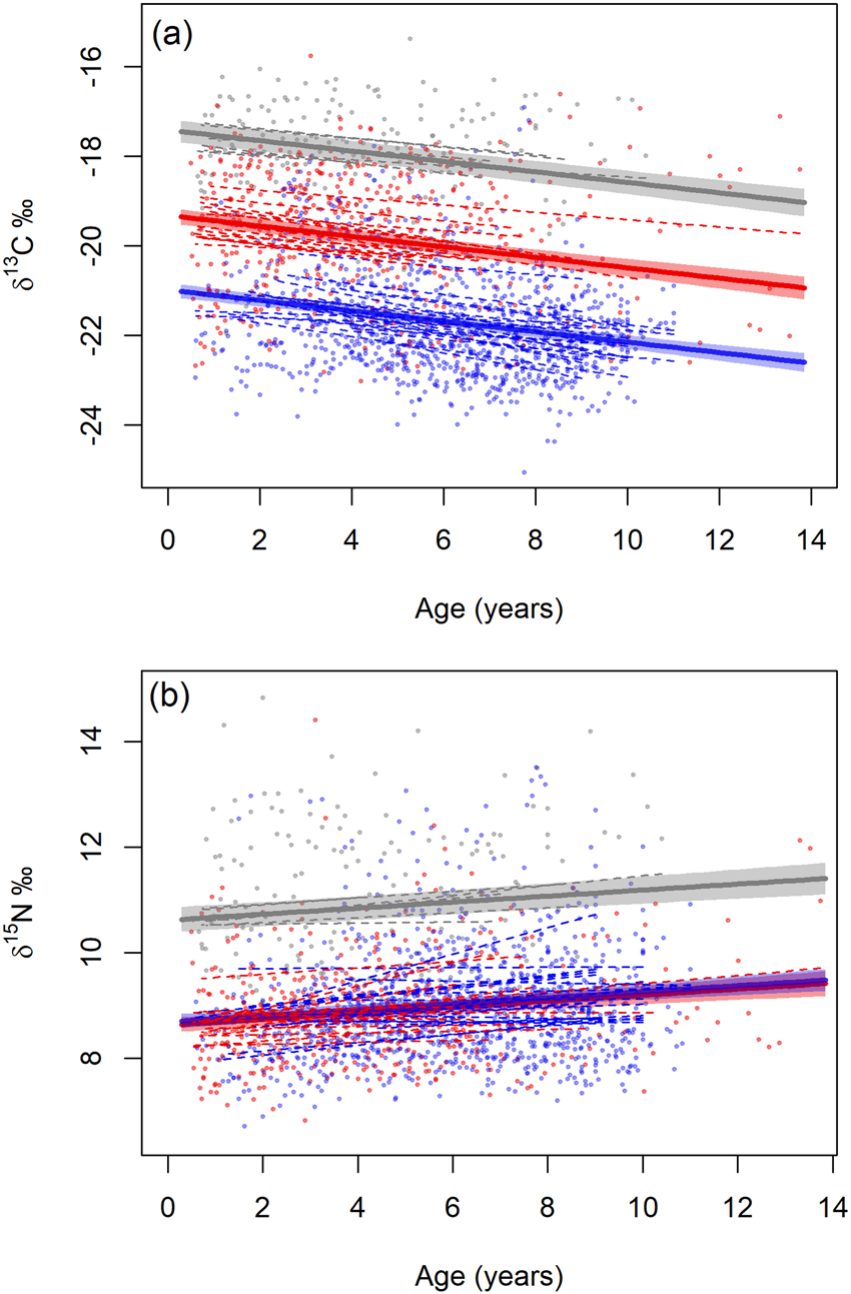
Best-fit linear mixed models explaining the change in (**a**) δ^13^C and (**b**) δ^15^N values along Antarctic fur seal whiskers with estimated age of males (blue) and minimum estimated age of females: female Group 1 (red; females with lower mean δ^13^C values than estimated δ^13^C value at the Polar Front) and female Group 2 (grey; females with higher mean δ^13^C values than estimated δ^13^C value of whiskers at the Polar Front). Points are isotope values of each whisker sample, dashed lines are fitted slopes explaining trend for each individual seal, bold lines are fitted trends for each group and shaded areas indicate standard error. Figure was created using R software (v3.6.1; https://www.R-project.org/).

The δ^13^C values along the length of whiskers, indicating changes in foraging distribution throughout ontogeny, were best explained by group (males, female Group 1 and female Group 2) and age (linear mixed model; likelihood ratio test LR = 76.4, p < 0.001; conditional R-squared = 49.3%). The δ^13^C values significantly differed among all three groups and values declined as seals aged (Table 1; Fig. 7a). Foraging distribution was highly generalised within the sample population, as the individual specialisation value was 0.89.

**Table 1:** Results of best-fit linear mixed models explaining the change in δ^13^C and δ^15^N values along the length of Antarctic fur seal whiskers: males, female Group 1 (females with lower mean δ^13^C values than estimated δ^13^C value of whiskers at the Polar Front) and female Group 2 (females with higher mean δ^13^C values than estimated δ^13^C value of whiskers at the Polar Front).

Fixed effects	Intercept Value	Degrees of freedom	p-value
**δ**^**13**^**C**			
Male (Intercept)	−21.61	1596	<0.001
Female Group 1	1.66	37	<0.001
Female Group 2	3.56	37	<0.001
Age	−0.30	1596	<0.001
**δ**^**15**^**N**			
Male (Intercept)	9.00	1596	<0.001
Female Group 1	−0.07	37	0.69
Female Group 2	1.93	37	<0.001
Age	0.15	1596	0.0038

The estimated portion of time that seals spent north of the Polar Front (based on δ^13^C values in sampled whisker segments and estimated δ^13^C value of whiskers when seals foraged at the Polar Front) was best described by group alone (Generalised linear mixed model; likelihood ratio test LR = 90.4, df = 2, p < 0.001). Female Group 2 spent the highest percentage of time north of the Polar Front (mean = 85.8% ± 8.7), followed by female Group 1 (mean = 32.6% ± 14.4) and males (mean = 2.6% ± 6.6).

The δ^15^N values along the length of whiskers, indicating changes in trophic position throughout ontogeny, were best explained by group and age (linear mixed model; likelihood ratio test LR = 7.72, p = 0.006; conditional R^2^ = 43.4%). Trends in δ^15^N values significantly increased with age for all groups and δ^15^N values significantly differed between males and female Group 2 but not between males and female Group 1 (Table 1; Fig. 7b). Individuals were slightly less generalised in trophic position than foraging distribution within the sample population, as the individual specialisation value was 0.76.

## Discussion

Niche partitioning plays a fundamental role in ecology by reducing competition for resources. This study revealed that the behaviour of Antarctic fur seals in the world’s largest breeding colony show distinct niche partitioning between sexes, throughout ontogeny and among individuals, which may help facilitate the high population density on South Georgia and the recovery of this population post-sealing. By analysing stable isotopes along whiskers we found strong support that (1) Males spend more time foraging in maritime Antarctica than females; (2) Males progressively spend more time foraging in maritime Antarctica during each annual cycle as they age; (3) Females demonstrate two main foraging strategies with 70% of females (female Group 1) mainly foraging south of the Polar Front and the remainder (female Group 2) mainly foraging to the north of it; (4) Migration strategies remained consistent between years. We discuss the potential underlying drivers of these findings and propose their key ecological consequences.

### Methodological considerations

Prior to interpreting results there are several caveats of our study to consider. The estimated whisker δ^13^C value when Antarctic fur seals foraged at the Polar Front was based on only one season of prey data and the proportion of each species’ contribution to the diet was unknown. Our estimated value was very close to −19‰: estimated by Cherel *et al*.^62^ and used by Kernaléguen *et al*.^63^ for Antarctic fur seals breeding at the Crozet Islands. It also closely aligned with isotope values in blood of seabirds foraging at the Polar Front from South Georgia^61^. However, the location and width of the Polar Front is not constant as a result of high variability in mesoscale meanderings, eddies and ring formations^61,65^. The value can therefore vary spatially and temporally and should only be considered as a broad indicator of foraging distribution. Baseline isotope ratios also change in time and space with sources of organic matter^66^. We could not account for these changes due to uncertainties inferring fine-scale foraging locations at set points in time from the isotope data, coupled with the lack of comprehensive isoscapes available for the geographical area (which vary seasonally and annually). The available isoscapes for the Antarctic Peninsula region revealed that δ^13^C values in particulate organic matter showed high annual variation (standard deviation of 2.9‰) in February each year between 2013 and 2016, but no general trend over time^67^. It is unlikely that trends in our results reflect changes in baselines, as there is currently no evidence of simultaneous trends in baseline isotope values in the Southern Ocean^68^.

The isotope data also presented additional sources of variation. In females, the exact point that whiskers were cut likely differed (within ~2 mm) among individuals, which may slightly affect the minimum ages of females. Since only minimum female age could be determined, we could not assess changes in isotope values with exact age. In males, we were unable to account for fasting during the breeding season. Fasting enriches *δ*^15^N values in organisms by 0.5‰ on average and has no signficiant effect on *δ*^13^C values^69^. However, male Antarctic fur seals will also forage during the breeding season^27^, so we were unable to determine the length of fasting by each male each year (as well as determine the enrichment in nitrogen, which may depend on seal age, size and health). Although this short period of fasting may have slightly increased the values of some data points, it is unlikely that this explains patterns in our results.

### Niche partitioning between sexes

Sexual segregation occurred along the spatial and temporal axis of the niche: male Antarctic fur seals had lower δ^13^C values than females, indicating they spent more time foraging further south in maritime Antarctica during each annual cycle than females, supporting hypothesis (1). This sexual segregation might be partially driven by breeding constraints, as females are restricted in the distance they can travel from pupping beaches when foraging to provision their pups, while males have no temporal or spatial limitations post-mating so can forage further afield^27,70^. Indeed, three Antarctic fur seal adult males tracked with satellite transmitters migrated south post-mating^44^ and young males marked with flipper tags have been re-sighted further south at Signy Island^43^.

There may also be a link between sexual size dimorphism and foraging niche. Lower δ^13^C values (indicating more southerly foraging) in males than females have also been reported in sexually dimorphic albatrosses and giant petrels breeding at South Georgia, but not in monomorphic burrowing petrels^61^. According to optimal foraging theory^71^ larger animals should prefer spatially clustered resources to decrease foraging costs^72^, as they have higher energetic needs^20,21^. For example, Albrecht *et al*. (2018) found that larger birds (of over 80 species) sampled along Mount Kilimanjaro foraged on plants with higher resource density than smaller birds. Male Antarctic fur seals require an estimated 3.8 tonnes of krill per year – twice as much as females^73^ and may therefore exploit the most productive areas available^48^. Krill density tends to be higher near the Antarctic Peninsula than South Georgia (1996–2016^74^) and there is large inter-annual variability in krill abundance and availability in the Scotia Sea^75^, which has been associated with sporadic declines in breeding success and population sizes of predators at South Georgia^76–78^. Males may exploit the greater density and predictability of krill near the Antarctic Peninsula to maintain a large body size. The Antarctic Peninsula and nearby islands appear to be less suitable for females to provision pups, as shown by the low numbers that breed there (e.g.^77,79,80^) relative to South Georgia.

Males and female Group 1 had similar δ^15^N values, indicating they competed for the same prey. They are likely opportunistic foragers, as the individual specialisation index for δ^15^N values showed greater generalisation than specialisation. Males potentially forage more successfully in the absence of females^42,44,70^, which reduces intra-specific competition. Spatial segregation between the sexes also occurs in grey seals, *Halichoerus grypus*, as males primarily use the continental shelf and females the mid-shelf, which the authors suggest acts to maximise fitness by reducing intersexual competition^81^. Shifting habitat, as opposed to diet, may be a more effective strategy to reduce competition^6^. By migrating south, male Antarctic fur seals could also reduce inter-specific competition with millions of breeding seabirds that congregate at South Georgia in summer. However, males likely increase spatial overlap with the krill fishery (largest fishery by tonnage in the Southern Ocean^82^), which operates at the Antarctic Peninsula in summer: a time when it is closed at South Georgia.

### Niche ontogeny

Individual niches are not fixed and can differentiate throughout an animal’s life^26,28,29^. We found that δ^15^N values and therefore trophic level of prey in male and female Antarctic fur seal whiskers gradually increased with age. Similar patterns have been documented with increasing body size in striped dolphins, *Stenella coeruleoalba*^50^ and great white sharks, *Carcharodon carcharias*^83^. This pattern may result from development of a larger mouth gape^84^, greater physiological capabilities (e.g. travel speed and aerobic dive limits^27^) and foraging experience, enabling larger individuals to handle larger higher-trophic-level prey with greater nutritional value. Alternatively, the increasing δ^15^N values may relate to changes in prey availability over time, such as declines in krill abundance as a result of climate change^74,85^. Indeed, Tarroux *et al*.^68^ attributed increasing δ^15^N values in blood and plasma of Antarctic fur seals breeding at Bouvetøya (from 1997–2015) to a shift in diet towards greater consumption of higher-trophic-level prey (replacing krill). This best explains trends in δ^15^N values along Antarctic fur seal whiskers at South Georgia, as δ^15^N values did not level when adults reached maximum body size.

Distinct ontogenetic niche shifts were present in males, supporting hypothesis (2). The δ^13^C values declined as males aged, indicating they progressively spent more time south during each annual cycle. This trend in δ^13^C values was also apparent in growth layers of male Antarctic fur seal teeth^86^. As males age they gain experience of the best foraging areas and may exploit abundant resources in maritime Antarctica to meet their growing energetic needs. A larger body size and better body condition will improve a male’s ability to gain and retain high quality territories with greater mating opportunities^42^. Larger body sizes also facilitate better heat retention^87^, enabling larger males to withstand the higher thermoregulatory costs of foraging in colder environments. Thermal tolerance also influenced sexual segregation in the most sexually dimorphic bird species: great bustards, *Otis tarda*^88^, and could be an overlooked factor driving sexual segregation.

Trajectories in ontogenetic niche shifts may differ between the sexes, as females reach maximum body size and become sexually mature earlier than males. Kernaléguen *et al*.^48^ found that female Antarctic fur seals breeding at Kerguelen had a similar isotopic niche to adult females by age 2. We could not assess whether this pattern occurred in females breeding at South Georgia, as we could only determine minimum female age since body length was a poor indicator of age (varying substantially among individuals; see Forcada & Hoffman^78^). However, trends in δ^13^C values along female whiskers suggest a more continuous change in isotopic niche, which requires further investigation.

Ontogenetic niche shifts can reduce intra-specific competition^11,89^, as only a subset of individuals will compete with one another at a specific time^29,90^. Sample sizes for males aged under 2 years were small (as a result of whisker wear). However, throughout the remainder of male Antarctic fur seal development, the greatest isotopic niche overlap (indicating competition for resources) occurred between female Group 1 and males aged 2–3 years. This overlap may result from similarities in body size^47^ and energetic requirements. Isotopic niche overlap gradually declined between the sexes as males grew and aged, showing progressive development of sexual segregation as ontogenetic shifts in males freed up resources available to females. This mechanism (which may occur in other sexually dimorphic species) substantially reduces intra-specific competition, which potentially increases female survival, reproductive rates and ultimately elevates population carrying capacity.

### Niche partitioning within females

Female Antarctic fur seals occupied a broader isotopic niche (SEA 1.5 × larger) than males, supporting hypothesis (3). This concurs with tracking studies whereby females migrated north to the continental shelf east of Patagonia^45,46^, south to the northern tip of Antarctic pack ice^45^, or remained within the vicinity of South Georgia^44–46^. However, stable isotope analysis allowed us to quantify foraging strategies into two main groups supporting hypothesis 4. The consistency of these foraging strategies within the two groups highlights the potential of familiarisation with a foraging area, allowing individuals to maximise net energy gain^46^.

Size dimorphism may be a cause or consequence of divergent foraging strategies, as female Group 1 and Group 2 differed in body size: a phenomenon also observed in female loggerhead turtles, *Caretta caretta*^91^. Size dimorphism could lead to distinct foraging strategies as larger animals are generally less susceptible to predation and have greater competitive abilities than smaller animals^91^. They tend to have lower stroke frequencies^92^ and lower mass-specific maintenance costs^93^, enabling them to migrate over greater distances than smaller animals using the same amount of energy. This size dimorphism could stem from early life e.g. size of tadpoles at metamorphosis affects size of adult frogs^94^. However, foraging strategies were consistent in female Antarctic fur seals and body mass and girth were the most important components in PC1 and PC2 respectively, suggesting body dimorphism was more likely a consequence of divergent foraging strategies. These strategies may initially develop when pups disperse after weaning and explore potential foraging sites^95^. Pups with bolder personalities could show greater exploration than shyer individuals, as documented in wandering albatrosses, *Diomedea exulans*^96^. Female Group 2 may discover better foraging opportunities north of the Polar Front, resulting in their larger body size. Indeed, loggerhead turtles that migrated further and foraged in more productive waters were significantly larger than other turtles – potentially investing more resources into growth^91^.

According to Schoener^3^, competition should result in overdispersion of niches. Marginal value theorem^97^ also predicts that an animal should leave a patch of resources and search for another when rate of resource gain falls under expected mean rate. Competitive interactions and low resource availability could have alternatively initiated the longer foraging trips by female Group 2. For example, at high population densities some three-spined stickleback, *Gasterosteus aculeatus*, become more opportunistic while others form novel dietary groups^98^ and when resource availability is limited some female parasitic wasps, *Pachycrepoideus vindemmiae*, immediately retreat from a resource patch, while others remain and are more involved in competitive interactions^99^. Female Group 2 may minimise intra-specific competition with males and Female Group 1, while gaining nutritional benefits that outweigh the energetic costs of locomotion^100^. Since Antarctic krill is almost exclusively distributed south of the Polar Front^101^, female Group 2 must predominantly feed on alternative species such as squid, myctophids and other fish – as found in the diet of Antarctic fur seals breeding at Kerguelen, Heard Island and Marion Island^49,102,103^. They are likely more susceptible to competition and interaction with an abundance of squid jiggers, longliners and benthic trawlers that operate in the South Atlantic. Prey consumption by female Group 2 (satisfying potentially 30% of the female population) could result in a greater impact on the South Atlantic marine ecosystem than previously realised.

## Conclusion

Stable isotope analysis (complimented with findings from short-term tracking studies) enabled us to reveal niche partitioning in the world’s largest Antarctic fur seal colony. Analysing stable isotopes along progressively growing tissues may be more practical, more cost-effective and less invasive than using short-term tracking methods alone^36^. We propose that the Antarctic fur seal colony breeding at South Georgia is generalist as a whole (indicated by individual specialisation indices), as seals could inhabit a range of environments, from warmer South Atlantic waters to colder Antarctic waters. However, the population is composed of more specialised strategies that may develop as a function of body size (with males experiencing a large range of body sizes and energetic requirements throughout ontogeny and female size differing according to foraging strategy). These strategies relax intra-specific competition, which may benefit population stability and carrying capacity, as well as the behavioural plasticity of the colony to adapt to changing environmental conditions. Intra-specific niche partitioning therefore has implications for ecology, evolution and conservation and is important to study in other species.

## Methods

### Ethics statement

The animal handling procedures in this study were reviewed and approved by the British Antarctic Survey Animal Ethics and Welfare Review Body (AWERB). The procedures adhered to the ASAB guidelines, ARRIVE guidelines and legal requirements of the South Georgia Government. The behavioural responses of adult females during restraint were predictable (given previous research conducted within the colony) and all efforts were made to minimise stress to individuals.

### Sample collection

Whiskers were collected from 30 freshly dead adult male and 25 live adult female Antarctic fur seals from September 2016 – February 2017 during the breeding season at Bird Island, South Georgia (54.010°S, 38.059°W). Dead males are regularly found ashore during the mating season: a reflection of the intense competition among males to gain access to females^104^. For males the two longest whiskers were pulled from both sides of the face, body length and girth measurements were recorded (fresh dead males only), an ID tag was applied to the skull, and after decomposition an upper canine was extracted from the jaw. An upper canine was extracted from an additional four dead males (34 teeth total), in which whiskers could not be obtained because of prolonged decomposition. Females rarely die ashore and no dead females were observed during this period. For each live female, the single longest whisker was cut from the right side of the face (as close to the skin as possible) during restraint (enabling whiskers to regrow). Females were weighed and body length, body span (nose to tail) and girth measurements recorded. The longest whisker (representing the longest period of growth) on the right side of the face was chosen from 20 randomly selected males and 20 randomly selected females for sample preparation.

### Sample preparation

Whiskers were washed with a sponge and Ecover detergent, transferred to a water bath for five minutes to remove contaminants (i.e. blood and dirt), then dried in an oven at 70 °C. Adhesive measuring tape was placed along each whisker and clear thin plastic positioned on the alternate side to ensure samples could be cut with accuracy and remained enclosed (to secure samples during cutting). Samples weighing a target weight of 0.7 mg were cut at the start of every 5 mm segment along the length of each whisker (most samples were 1–2 mm in length). Each 5 mm segment represented approximately 1.5–2.8 months of whisker growth based on growth rates calculated by Kernaléguen *et al*.^48^. Samples were removed from the tape using tweezers and placed in glass vials with a 2:1 chloroform:methanol solvent to remove any lipids and tape residue stuck to the whisker to leave clean keratin^105^. Samples were dried in a fume hood overnight then weighed into 3 × 5 mm tin capsules for mass spectrometry. Total sample sizes were 1011 for males and 642 for females.

### Mass spectrometry

Tin capsules were loaded into the autosampler of an Elementar (Hanau, Germany) Pyrocube Elemental Analyser, which converted carbon and nitrogen in the samples to CO_2_ and N_2_ gases. The ratios of carbon and nitrogen isotopes in these gases were measured on a Thermo-Fisher-Scientific (Bremen, Germany) Delta XP Plus Isotope-Ratio Mass Spectrometer. The internal reference materials (mean ± SD) were GEL (gelatin solution, δ^13^C= −20.09 ± 0.19‰, δ^15^N= 5.59 ± 0.12‰), ALAGEL (alanine-gelatine solution spiked with ^13^C-alanine, δ^13^C = −8.69 ± 0.17, δ^15^N = 2.22 ± 0.08‰), and GLYGEL (glycine-gelatine solution spiked with ^15^N-alanine, δ^13^C = −38.35 ± 0.13‰, δ^15^N = 23.19 ± 0.22‰), each dried for two hours at 70 °C. Four USGS 40 glutamic acid standards^106,107^ were used as independent checks of accuracy. Delta values were corrected for instrument drift (changes in isotopic composition of gases through the mass spectrometer) and linearity (variability in sample masses). Stable isotope ratios were expressed in parts per thousand (‰) deviation from the international standards (Vienna Pee Dee Belemnite for carbon and AIR for nitrogen), according to the equation:

$${\rm{\delta }}{\rm{X}}=[({\rm{Rsample}}/{\rm{Rstandard}})-1]$$

where X is ^15^N or ^13^C and R is the corresponding ratio (^15^N/^14^N) or (^13^C/^12^C). Stable isotope ratios were reported as δ^13^C values for carbon and δ^15^N values for nitrogen.

### Age determination

Each male seal was aged by three readers by counting external growth ridges on the extracted upper canine. These ridges, formed from annual deposition of dentin, are prominent in male Antarctic fur seals^108^. The modal ages were assumed for each individual. Precision in age determination was estimated using the IAPE, as described by Beamish and Fournier^109^ according to equation:

$$IAPE=\frac{1}{N}\,\mathop{\sum }\limits_{j=1}^{N}\,[\frac{1}{R}\,\mathop{\sum }\limits_{i=1}^{R}\,\frac{|{X}_{ij}-\,{X}_{j}|}{{X}_{j}}]\times 100$$

N is the total number of individuals aged, R is the number of times each individual is aged, and X_*ij*_ is the *i*th age determination of the *j*th individual. A smaller IAPE indicates more precise age determinations. Females were first aged according to their measured body length by extrapolating age from a modelled body length-to-age curve (Fig. 1d in Forcada & Hoffman^78^). Age was not estimated for five females, as body lengths exceeded modelled lengths in the growth curve.

Whisker growth rates were calculated using wavelet analysis, which can assess the degree of periodicity in stable isotope values along the length of whiskers (as described by Kernaléguen *et al*.^63^). For each seal whisker the wavelet transform was applied and a power spectrum produced using the ‘WaveletComp’ package^110^ in R^111^. The power spectrum specified significant periodicity in δ^13^C values, which were used to reconstruct the original time series by ‘denoising’ the series and retaining the smooth components. These reconstructed time series were used to calculate the growth rate of each whisker, assuming oscillations corresponded to annual migrations. Since whiskers of four females and four males demonstrated no clear periodicity in δ^13^C values, the average growth rates of all female and male whiskers were applied respectively for these individuals. For each of the 20 male seals, age was estimated along the length of the whisker using whisker growth rate and seal age (obtained from external growth ridges in canine) by back-tracking along the whisker (facial end to whisker tip). This method was repeated for the 20 females using the estimated ages obtained from body length. However, age estimates are highly variable with body length^78^ and body length substantially underestimated female age at capture (by 3.5 years on average) according to female age determined by whisker growth rates. Whisker growth rates were considered more reliable (since oscillations in δ^13^C values likely correspond to annual migration patterns) and were used alone to determine minimum female age along the length of each whisker for following analyses. Exact female ages could not be determined as a result of whisker wear/breakage at the tips and because whisker growth rates were not definite. Characteristic peaks in δ^15^N values at the tips of six female whiskers likely corresponded to suckling patterns, suggesting these whiskers had not broken. In these cases, δ^15^N peaks were lined up and age was estimated along the whisker (whisker tip to facial end) using the calculated whisker growth rates.

### Data analysis

To broadly determine Antarctic fur seal foraging distribution using stable isotope values we approximated the δ^13^C value for whiskers when seals foraged at the Polar Front (convergence between cold Antarctic waters and warmer sub-Antarctic waters). We first determined the TDF for Antarctic fur seal whiskers using the SIDER package^112^ in R^111^. SIDER estimates the TDF for a particular consumer and tissue (in which controlled feeding studies are impractical) using a phylogenetic regression model, fitted using Bayesian inference to a compiled dataset of TDF values of phylogenetically and ecologically related species^112^. We secondly added this TDF to the average δ^13^C value of prey items (myctophids and krill) collected at two locations at the Polar Front (50.0632°S, 34.0287°W and 49.9357°S, 34.2078°W) during research cruise JR200 (British Antarctic Survey) in Autumn 2009. The resulting δ^13^C value for Antarctic fur seals whiskers when seals foraged at the Polar Front was then overlayed on stable isotope bi-plots to assess differences in foraging distributions between and within the sexes. Since bi-plots revealed two isotopically distinct groups of females, with average δ^13^C value of each individual falling lower or higher than the estimated δ^13^C value of whiskers when seals foraged at the Polar Front, females were split into two groups (female Group 1 and female Group 2 respectively) for subsequent analyses. To test whether body morphology significantly differed between female Group 1 and Group 2, we ran a PCA on body mass, length, span and girth measurements, and used the output from PC1 and PC2 as separate response variable in Welch’s t-tests.

To compare male and female isotopic niche areas we used the SIBER package in R^43,113^ to calculate SEAs (encompassing 40% of data points) according to maximum-likelihood estimation, as well as SEA_*B*_s according to Bayesian inference to account for uncertainty in ellipse areas. The Bayesian Standard Ellipse Areas were calculated using 100,000 posterior draws and the mode and 95% credibility intervals were reported. The proportions of overlap between male and female prediction ellipse areas, and between female Group 1 and Group 2 ellipse areas, were calculated to quantify isotopic niche differentiation between these groups – first using maximum-likelihood estimation, then using Bayesian inference with 100,000 posterior draws. This method was repeated to quantify ontogeny of isotopic niche differentiation on an annual scale in males (from 0 to 11 years of age) and to assess overlap among these niches and overall SEAs of female Group 1 and 2.

Since sexual segregation can also occur along the δ^13^C and δ^15^N axes separately, δ^13^C and δ^15^N values were used as separate response variables in linear mixed models^36^. We tested whether δ^13^C and δ^15^N values significantly differed among males, female Group 1 and female Group 2 using a global model, refined by backward-stepwise deletion and likelihood ratio tests using the ‘nmle’ package^114^ in R. Each global model included group (males, female Group 1 and female Group 2), age, and the interaction between group and age as fixed effects. Age was used as a random intercept and slope to account for variability in δ^13^C and δ^15^N values among individuals as they aged and a corARMA structure (p = 2, q = 0) was used to account for temporal autocorrelation in residuals. We additionally tested whether males, female Group 1 and female Group 2 differed in time spent north of the Polar Front as they aged by calculating the proportion of time spent north of the Polar Front (based on whether δ^13^C values exceeded the estimated δ^13^C value of whiskers when seals foraged at the Polar Front), which was used as the response variable in a generalised linear mixed model with a Beta error family, refined as above. Individual specialisation indices were determined, corresponding to the average similarity among individuals and the population^115^. The variance components were partitioned from each best-fit model and the within individual component (WIC) was divided by trophic niche width (TNW). An individual specialisation index of 0 indicates individuals are complete specialists, while a value of 1 indicates individuals use the whole range of the sample population’s niche^116,117^. All results were reported as means plus standard deviations unless stated.

## Supplementary information

Supplementary Information.
