# Supplementary material for: Intra-specific Niche Partitioning in Antarctic Fur Seals, Arctocephalus gazella
Source: Sci Rep. 2020 Feb 24;10:3238. doi: 10.1038/s41598-020-59992-3 (PMC7039921; doi:10.1038/s41598-020-59992-3)
Supplement: Supplementary file 1 — Supplementary Information. [file 41598_2020_59992_MOESM1_ESM.pdf]

Supplementary Material

Intra-specific Niche Partitioning in Antarctic Fur Seals, *Arctocephalus gazella*

Kayleigh Jones[1,2], Norman Ratcliffe[1], Stephen C. Votier[2], Jason Newton[3], Jaume Forcada[1], John Dickens[1], Gabriele Stowasser[1], Iain J. Staniland[1]

[1]British Antarctic Survey; [2]University of Exeter; [3]Scottish Universities Environmental Research Centre

Supplementary Figure S1. Oscillations in $\delta^{13}C$ values along the length of whiskers (from the distal to facial end) in (a–c) three male Antarctic fur seal whiskers (IDs = w8315, w8580 and w8675 respectively) and (d–f) three female whiskers (IDs = w8868, w8858 and w8870 respectively). Points are $\delta^{13}C$ values of samples taken every 5 mm along the length of each whisker and lines join these points. Male age was determined by external growth ridges on canines and minimum female age was determined by whisker growth rates.

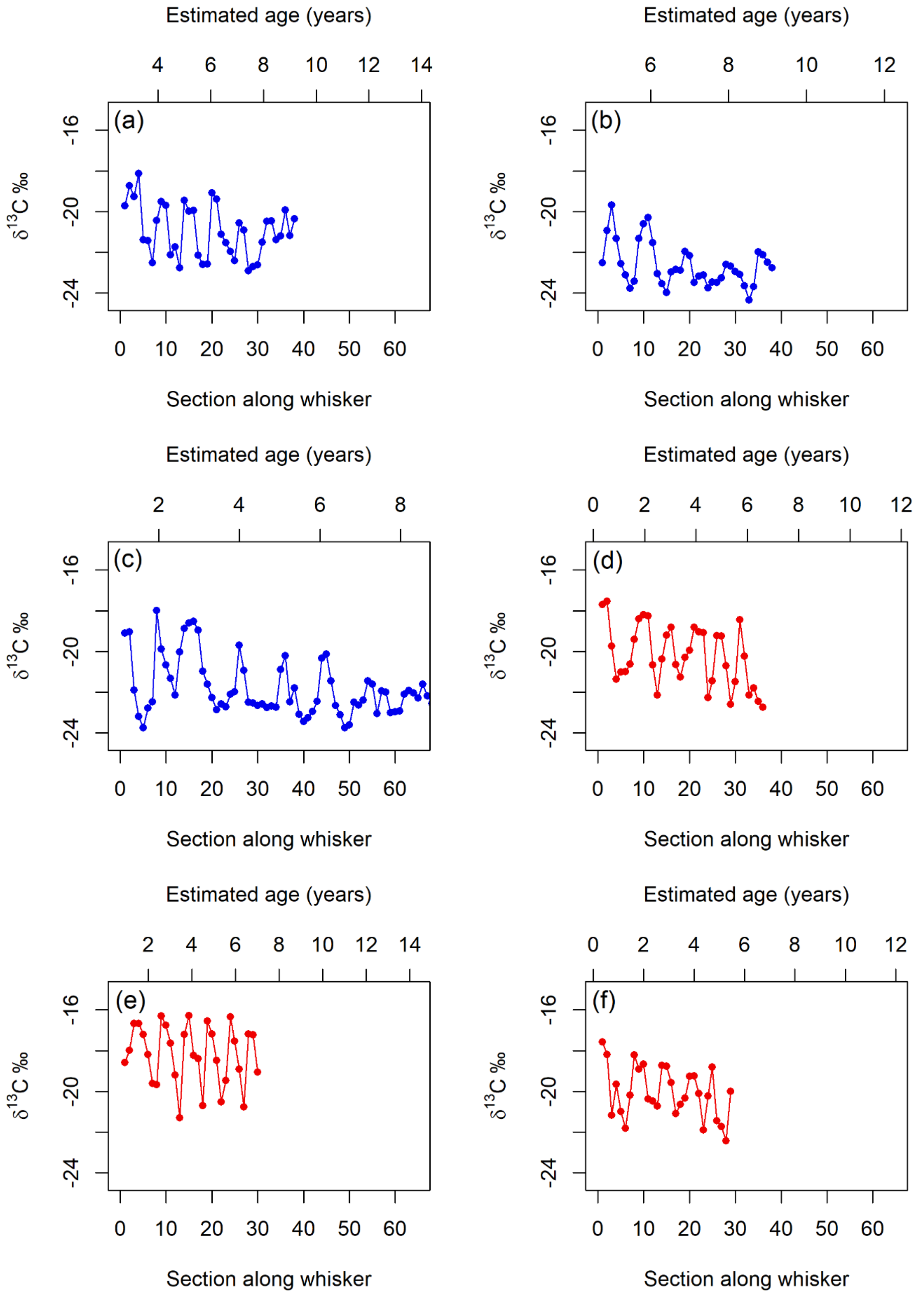

Supplementary Figure S2. Bi-plot showing the mean (points) and standard deviation (lines) of $\delta^{13}C$ and $\delta^{15}N$ values of each prey species (Antarctic krill, *Euphausia superba*, and myctophids (remainder of species)) collected at two sample locations at the Polar Front (50.0632° S, 34.0287° W and 49.9357° S, 34.2078° W) in Autumn 2009.

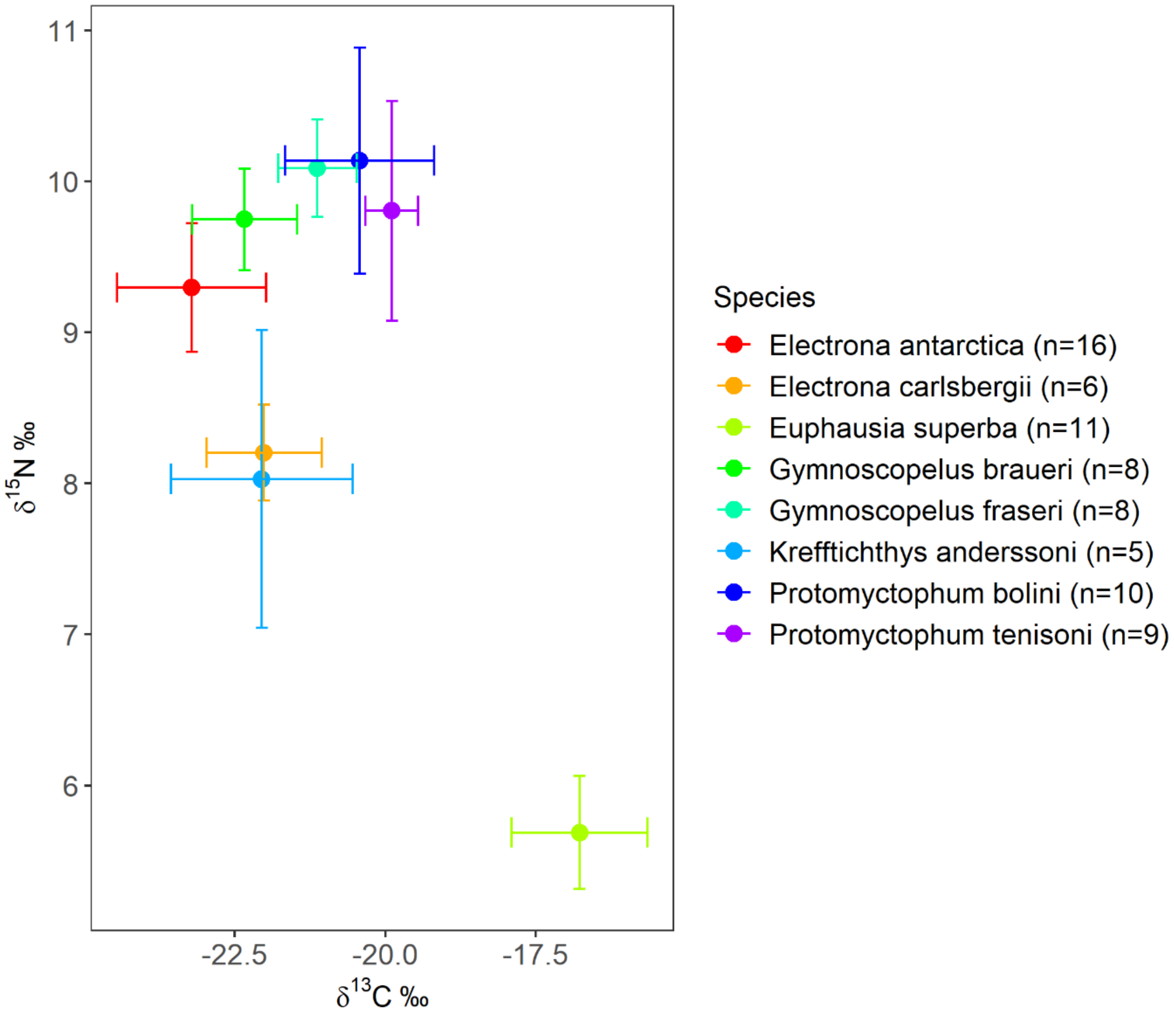

Supplementary Table S3. Length of the longest whisker on the right side of the face of 20 live adult female Antarctic fur seals, as well as body length, estimated age based on body length alone, minimum estimated age based on oscillations in $\delta^{13}C$ values along each whisker and minimum and maximum $\delta^{13}C$ and $\delta^{15}N$ value along each whisker. Asterisks indicate that a weaning signal (characterised by high $\delta^{15}N$ values followed by a drop) was present at the distal end of the whisker.

ID	Whisker length (cm)	Body length (cm)	Estimated age using body length	Minimum age using oscillations in $\delta^{13}C$ (%)	Min $\delta^{13}C$ (%)	Max $\delta^{13}C$ (%)	Min $\delta^{15}N$ (%)	Max $\delta^{15}N$ (%)
w8851	17.4	130.5	Unknown	7.7	-22.18	-17.98	6.83	10.46
w8852	16.0	128	Unknown	7.4	-21.80	-15.76	7.72	14.42
w8854	14.4	118.75	3	5.6	-22.21	-18.18	7.35	10.39
w8855	13.3	126	6	7.0	-19.32	-16.38	8.80	12.63
w8856	11.4	119.5	3	4.0	-21.94	-16.72	8.27	13.08
w8857	16.6	128.5	Unknown	10.4	-22.04	-16.06	8.80	14.84
w8858	15.0	123	4	6.8	-21.31	-16.28	8.36	12.69
w8859	16.5	120.25	4	6.6*	-23.01	-17.87	7.79	11.51
w8860	13.0	134	Unknown	8.6	-19.02	-16.51	8.27	12.74
w8862	16.6	125.5	6	7.6	-22.70	-17.92	7.44	10.03
w8863	30.6	124.5	5	13.7	-22.60	-16.62	7.23	12.13
w8864	16.8	121.25	4	8.2	-20.35	-15.38	8.53	14.32
w8865	25.7	131.75	Unknown	10.2	-22.57	-17.49	7.04	10.81
w8867	12.9	126	5	5.6*	-22.74	-17.98	7.61	10.68
w8868	18.0	117	3	6.4*	-22.75	-17.53	7.61	12.26
w8869	15.5	122	4	6.8*	-22.14	-17.75	7.12	10.74
w8870	14.6	120.75	4	5.3*	-22.43	-17.58	7.37	10.93
w8871	14.3	118	3	6.1*	-22.62	-17.74	7.49	12.42
w8874	11.0	119.25	3	6.0	-22.23	-18.12	7.66	11.32
w9150	16.1	112	3	8.8	-22.20	-16.87	7.31	11.19

Supplementary Table S4. Length of the longest whisker on the right side of the face of 20 dead adult male Antarctic fur seals, as well as body length, estimated age based on external growth ridges in the upper canine and minimum and maximum $\delta^{13}C$ and $\delta^{15}N$ value along each whisker. Body length could not be measured for decomposed males.

**ID**	**Whisker length (cm)**	**Body length (cm)**	**Estimated age using canine**	**Min $\delta^{13}C$ (%)**	**Max $\delta^{13}C$ (%)**	**Min $\delta^{15}N$ (%)**	**Max $\delta^{15}N$ (%)**
w8278	15.6	181	10	-23.30	-20.86	7.93	11.11
w8313	19.0	172	8	-22.92	-18.13	7.24	11.70
w8328	41.5	169	9	-23.47	-19.53	7.11	10.26
w8429	31.0	180	9	-23.02	-19.43	7.22	10.25
w8522	26.3	181	10	-23.47	-19.23	7.82	11.79
w8525	28.2	182	9	-23.09	-19.08	7.37	10.82
w8580	19.1	173	8	-24.36	-19.67	7.79	10.36
w8610	17.2	177.5	9	-25.05	-20.56	7.75	12.98
w8614	30.3	-	9	-23.80	-18.48	7.30	11.74
w8627	22.6	183	8	-23.21	-20.23	7.84	11.65
W8640	24.7	168	9	-23.67	-19.00	7.59	10.81
w8675	36.5	177	8	-23.75	-17.98	6.72	10.39
w8730	22.2	179	8	-23.16	-19.00	7.62	11.07
w8737	22.1	178	9	-23.21	-20.07	7.94	10.34
w8787	30.6	182	8	-23.37	-19.30	7.70	13.07
w8820	23.5	-	8	-24.35	-18.73	7.56	10.34
w8821	34.2	-	8	-23.00	-16.91	7.59	13.52
w8853	25.1	-	9	-23.38	-19.83	7.51	10.01
w8901	28.8	-	10	-23.32	-19.61	6.90	12.98
w9034	16.5	192	8	-23.33	-18.34	8.22	10.91

Supplementary Figure S5. Relationship between Principal Component 1 (explaining 73.9 % of variability) and Principal Component 2 (explaining 18.2 % of variability) of morphology measurements taken from 14 female Antarctic fur seals (red; Group 1; those with lower mean $\delta^{13}C$ values than estimated whisker $\delta^{13}C$ value at the Polar Front) and 6 female Antarctic fur seals (grey; Group 2; those with higher mean $\delta^{13}C$ value than estimated whisker $\delta^{13}C$ value at the Polar Front).

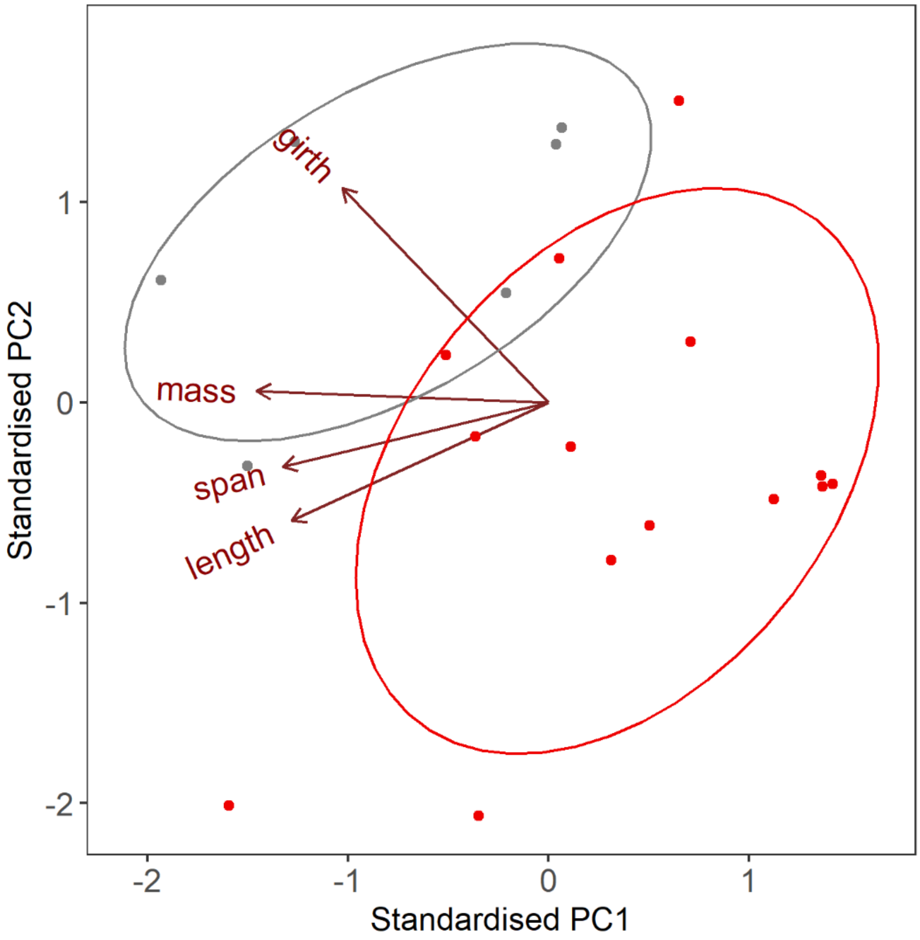

Supplementary Table S6. Standard Ellipse Areas (SEAs) and Bayesian Standard Ellipse Areas (SEA_Bs) quantifying the isotopic niches of 20 male Antarctic fur seals throughout ontogeny using $\delta^{15}N$ and $\delta^{13}C$ values along whiskers.

Male age (years)	SEA	SEA_B mode	SEA_B 95% confidence interval	No. of males	No. of male whisker samples
**0.5 – 1**	0.640	0.562	0.177 – 2.123	2	6
**1 – 2**	5.710	5.610	3.947 – 7.745	4	17
**2 – 3**	3.800	3.756	2.951 – 4.837	10	47
**3 – 4**	3.611	3.601	2.988 – 4.328	17	92
**4 – 5**	3.310	3.311	2.774 – 3.880	19	128
**5 – 6**	3.916	3.898	3.286 – 4.629	19	131
**6 – 7**	3.256	3.259	2.745 – 3.824	20	140
**7 – 8**	3.813	3.814	3.219 – 4.506	20	141
**8 – 9**	2.573	2.576	2.185 – 3.002	20	145
**9 – 10**	1.914	1.873	1.525 – 2.403	12	87
**10 – 11**	1.488	1.421	0.922 – 2.373	6	42

Supplementary Table S7. Standard Ellipse Areas (SEAs) and Bayesian Standard Ellipse Areas (SEA_Bs) quantifying the overlap in isotopic niches of 20 males with 14 females in female Group 1 (those with lower mean $\delta^{13}C$ values than estimated whisker $\delta^{13}C$ value at the Polar Front) throughout male ontogeny using $\delta^{15}N$ and $\delta^{13}C$ values along whiskers.

Male age (years)	SEA overlap	SEA_B overlap mode	SEA_B overlap 95% confidence interval	No. of males	No. of male whisker samples
**0.5 – 1**	0	0	NA	2	6
**1 – 2**	14.832	14.203	6.586 – 22.212	4	17
**2 – 3**	30.785	27.687	22.562 – 39.125	10	47
**3 – 4**	22.600	21.611	15.138 – 27.873	17	92
**4 – 5**	10.321	10.258	5.959 – 15.371	19	128
**5 – 6**	5.675	5.792	1.857 – 9.473	19	131
**6 – 7**	0.00154	0.001	0.000 – 0.224	20	140
**7 – 8**	0.000	0.001	0.000 – 0.021	20	141
**8 – 9**	0.000	0.000	NA	20	145
**9 – 10**	0.000	0.000	NA	12	87
**10 – 11**	0.000	0.000	NA	6	42
